# Moral judgement and decision-making: theoretical predictions and null results

**DOI:** 10.1038/s41598-023-34899-x

**Published:** 2023-05-11

**Authors:** Uri Hertz, Fanli Jia, Kathryn B. Francis

**Affiliations:** 1 Department of Cognitive Sciences, University of Haifa, 3498838 Haifa, Israel; 2 Department of Psychology, Seton Hall University, South Orange, NJ 07079 USA; 3 School of Psychology, Keele University, Keele, Staffordshire ST5 5BG UK

**Keywords:** Psychology, Human behaviour

## Abstract

The study of moral judgement and decision making examines the way predictions made by moral and ethical theories fare in real world settings. Such investigations are carried out using a variety of approaches and methods, such as experiments, modeling, and observational and field studies, in a variety of populations. The current Collection on moral judgments and decision making includes works that represent this variety, while focusing on some common themes, including group morality and the role of affect in moral judgment. The Collection also includes a significant number of studies that made theoretically driven predictions and failed to find support for them. We highlight the importance of such null-results papers, especially in fields that are traditionally governed by theoretical frameworks.

## Introduction

The study of moral judgement and decision making examines the way people behave and react to social and moral dilemmas. Moral and ethical theories usually provide the foundation for such efforts, providing important constructs and definitions, and even suggesting hypothetical experimental designs. A good example is the differentiation between deontological and utilitarian basis of moral action selection. Characteristically utilitarian approaches look at the overall benefit of each action, while characteristically deontological approaches set principles, prohibiting some actions regardless of their ultimate outcome. Both approaches provide predictions for moral decisions and use hypothetical scenarios such as personal versus impersonal trolley-type problems to illustrate the different predictions. In recent years researchers have been putting such theories to the test in a variety of experimental designs and populations. Translating theoretical hypotheses and constructs to an experimental paradigm or an operational prediction is not trivial. Participants’ individual traits and their cultural and societal context introduces variability and nuances to ethical theories. In addition, the technical need to build a robust and reliable experimental design, which can be evaluated using statistical tools, leads researchers to adopt experimental designs from different fields, such as economics and cognitive psychology.

## Common themes in the collection

The current Collection invited works that employ a variety of paradigms and analyses tools to experimentally test predictions of moral judgement and decision-making. At the time this Editorial is written, the Collection covers several themes in moral judgment and decision making with the research included using different experimental approaches.

One common theme regards the deontological-utilitarian response differences mentioned above, studied from different approaches. One study examined whether people tend to trust deontological decision makers more than utilitarians^1^, another looked at the persuasive effect of deontological and utilitarian messages^2^, and yet another examined the way depression affected utilitarian and deontological aspects of moral decisions^3^. Like other works in this Collection, these include experimental designs that relied on vignettes, describing such moral dilemmas as the footbridge problem. To study how trust inference of moral decision-makers is moderated by several contextual factors , Bostyn et al.^1^ used a behavioral game theory task, the trust game, where participants endow some of their money to a trustee in the hope that they will reciprocate, and the amount indicates their level of trust. Other studies in this Collection used monetary transactions as a proxy for cooperation and trust, using trust games^4^, the common-goods game^5,6^, decisions under risk^7^, variations of the dictator game where participants split money with others^8,9^ and paradigms in which participants gain money from harm to others^10,11^. The use of such different approaches in the study of the same topic is important, as it allows evidence to converge across different studies, each with its own weaknesses and strengths.

Another common theme is the move beyond the single decision maker to examine group and collective effects on moral judgement and decisions. Two studies examined the effect of diffusion of moral responsibility and causality. Hansson et al.^9^ studied the hypothesis that voting may lead to diffused responsibility, and through it to more selfish and immoral behavior. Keshmirian et al.^12^ studied the moral judgment of individuals that performed moral transgressions against their own or in group, where causal responsibility may appear to be diffused. Interestingly, no evidence was found for diffusion of responsibility on moral behavior, while diffusion was found to affect moral judgment and punishment. In another study, norms and knowledge of other people’s actions were found to affect risk-based decisions concerning others’ wellbeing^7^.

At the time of writing this editorial, the Collection included studies that examine other aspects of model decisions. Hoeing et al.^5^ examined whether political ideology affects moral decisions regarding money allocations, and Holbrook et al.^13^ examined cultural differences in moral parochialism judgments. Moreover, Krupp and Maciejewski^14^ discussed the evolutionary aspects of self-sacrifice in the context of interactions between sedentary actors and kinship, and Atari et al.^15^examined corpora of everyday discussions and evaluated how many of these are devoted to morality. These works indicate that moral judgment and behavior should be studied not only at the individual level, but also as a collective phenomenon; an emerging property of groups of interacting individuals. Others examined the effects of individual traits and emotions on moral decisions. Yin et al.^3^ examined the levels of emotional and cognitive processes of depression on moral judgement. Du et al.^11^ demonstrated mindfulness training could prevent moral preference decline over time without changing emotional regulation strategies. Diaz and Prinz^16^ found that level of emotional awareness (such as alexithymia) played an important role in moral evaluation while controlling reasoning.

## The rise of null-results in experimentation of moral theories

An important common characteristic in this Collection is the report of null-results. A number of studies took up an important theoretical question, used pre-registered experimental designs and sample sizes to tackle it, and reported no evidence supporting their initial hypothesis. For example, Hansson et al.^9^ examined whether responsibility diffusion lead voting crowds to behave more selfishly than individuals in two preregistered experiments and found no evidence for such an effect. Bahník and Vranka^8^ studied the effect of moral licensing on bribe taking in a preregistered study, hypothesizing that avoiding a small bribe may lead to increased likelihood to take a larger bribe later, and did not find evidence for such moral licensing effect. Cabrales et al.^4^ hypothesized that in a trust game, time constraints may push trustees to be more generous, and therefore that knowledge about trustee’s time constraints may make participants more likely to trust them. In three experiments, they found no evidence for this effect. Hoenig et al.^5^ examined the cooperation levels of left-leaning and right-leaning individuals and found that left-leaning individuals tended to cooperate more only on decisions that involved equality and did not differ from right-leaning individuals on decisions where outcomes were non-equally dispersed. Bocian et al.^17^ manipulated when moral information should be presented, expecting that the manipulation would moderate the impact of liking bias on moral judgment. However, the results of their preregistered study 2 did not support this hypothesis. Finally, in a registered report, Bostyn et al.^1^ tested the way contextual features affect trust in deontological and utilitarian decision makers, following Everett et al.^18^. They found no evidence of an overall effect that people trust deontological decision makers more than utilitarian ones.

These results, obtained by studying thousands of participants on multiple platforms and using multiple experimental designs, pose an important contribution to the literature on moral judgement and decision making. While all studies relied on sound theoretical principles, their null results help delineate the limits of these theories’ predictive power. As we argued above, carrying out experiments involves making practical decisions about populations, experimental designs and manipulations, and statistical analyses. This means that experimenters must deal with more nuanced, complex, and context-dependent effects than the more abstract and context-independent settings in which theoretical predictions are made. Experimental evidence, both in support of and against theoretical predictions, is important in the process of refining key moral theories; enabling researchers to investigate under which circumstances they operate as well as their limits.

Traditionally, null-results are less likely to be published, either due to the editing and review process, but also because of the self-censoring processes by which authors are less likely to finalize and submit for publication these projects^19^. As demonstrated here, the process of preregistration, and especially registered reports, ensures that these projects are published and shared with the relevant academic communities. This is important, as null-results are informative and can greatly contribute to the literature and theoretical development of the field. It is also important to highlight these results and encourage other researchers to experimentally test their theoretical predictions without fearing the lost cost of obtaining no evidence to support them. This is especially important in the field of moral decision making, which heavily relies on moral and ethical theory, and where experimentation can greatly inform broad societal problems.
